# The impact of partner’s behaviour on pregnancy related outcomes and safe child-birth in Pakistan

**DOI:** 10.1186/s12884-023-05814-z

**Published:** 2023-07-14

**Authors:** Muhammad Atif, Muhammad Farooq, Muhammad Shafiq, Gohar Ayub, Muhammad Ilyas

**Affiliations:** 1 Department of Statistics, University of Peshawar, Peshawar, Pakistan; 2 Institute of Numerical Sciences, Kohat University of Science and Technology, Kohat, Pakistan; 3 Department of Mathematics and Statistics, University of Swat, Mingora, Pakistan; 4 Department of Statistics, University of Malakand, Chakdara, Pakistan

**Keywords:** Antenatal care, Postpartum, Pregnancy, Child-birth, Partner, Labor

## Abstract

**Background:**

Pakistan is one of the nations with the worst statistics for pregnancy-related outcomes. Health programmes in underdeveloped nations frequently ignore the role of partners in maternal health, which is a crucial contributing factor in these worst situations. This research study aims to explore the role of husbands in maternity care and safe childbirth in Pakistan.

**Methods:**

The data for this study comes from the Pakistan Maternal Mortality Survey 2019. The *k*-Modes clustering algorithm was implemented to generate clusters from the dataset. Cluster profiling was used to identify the problems in pregnancy-related outcomes in cases where women live away from their partners. The chi-square test and logistic regression model were fitted to identify the significant factors associated with women’s health and safe childbirth.

**Results:**

The finding of the study reveals that the partner’s support during and after pregnancy plays a vital role in maternal health and safe child-birth. It was revealed that the women living away from their partners have certain health problems during pregnancy. These problems include Vaginal bleeding, Excessive vomiting, Chest pain, Cough, High B.P, Excessive weight gain, Body aches, Swelling of feet, and Swelling of the face. This also leads to complications and health problems in the postpartum period. Due to a lack of antenatal care from the spouse during pregnancy, the women who lived away from their partners lost their pregnancies.

**Conclusion:**

The study concludes that the husband’s emotional and financial support substantially impacts the overall health of expecting mothers and the safety of delivery in Pakistan. Given its potential advantages for mother and child health outcomes, male engagement in health education must be acknowledged and addressed. The finding of the study is of immense importance, as it guides the policymakers to arrange various awareness programs for the male partners to support their pregnant spouse and provide proper antenatal care.

## Background

Pregnancy is one of the most exciting and happy moments in the couple’s lives; however, it might also be challenging. As she becomes used to their growing baby, the pregnant partner may undergo significant emotional and physical changes. Although most women perceive pregnancy as a happy, beautiful experience, doubts and worries frequently arise. They feel exposed during pregnancy, and as a result, they grow more dependent on their relationship, and the help of friends and family becomes more critical [1, 2]. Maryam et al. [3] showed that the spouse’s support affects the emotional and physical health of a woman during pregnancy.

By responding to problems, requesting medical attention, covering travel expenses, and managing household resources, men can reduce several risk factors of maternal mortality [4, 5]. In addition, research has shown that men’s involvement in pregnancy outcomes significantly helps lowering teenage pregnancy, maternal and infant mortality, risky abortions, and fertility rates. Although, the care a mother receives before, during, and immediately after childbirth is crucial to the health and life of the mother and infant. However, in many countries, husbands’ contributions to women’s health and delivery are frequently neglected. Men, for instance, have minimal experience with or awareness of maternal health in South Asian context [6, 7].

The ignorance on the part of the male partner can influence several factors. These factors include delays in the decision of availing health care facilities, attaining antenatal care, and availing health care facilities before, during, and after pregnancy. These are the major contributing factor to raised maternal mortality and child death ratio [8]. However, awareness regarding men’s contributions to preserving pregnancy outcomes has grown recently [9, 10].

Naushin [11] argues that socioeconomic and demographic factors substantially affect the decision-making on reproductive behaviour. For example, women’s empowerment may help explain why women want fewer children and use contraception more regularly. In addition, the rising involvement of Pakistani women in family decision-making affects fertility outcomes, which are key policy objectives.

Pakistan has achieved a decline in maternal and neonatal mortality rate since 1990, yet there has been a small gain in these outcomes comparatively to other countries [12]. Stillbirth and pregnancy lost have rarely been addressed and remains under-reported in Pakistan [13]. Causes for these poor outcomes potentially include women of reproductive age being poorly educated, anaemic, and delivering a high percentage of preterm and low-birth weight babies [14]. In Pakistan, males are crucial in the decision-making process for family matters, particularly when it comes to reproduction [15]. Therefor, it is important to explore the role of husband in providing Antenatal care and ensure safe childbirth. Several studies have been conducted to discuss this issue, but all of these studies consider socio-demographic factors. To our knowledge, so far, no study has examined the role of a husband in antenatal care before, during, and after his wife’s pregnancy. Therefore, this study aimed to assess male involvement in providing antenatal care to their wife in Pakistan. The objective of this research is to examine the role of the husband’s attitude on the physical health of pregnant women. This will help understand males’ awareness and serve as a guide for primary care physicians and reproductive health targeted programs.

## Literature review

Maryam et al. [3] discovered that emotional and financial support from partners can significantly reduce stress levels in expectant mothers. They also observed that when partners actively participated in prenatal meetings, it led to a decrease in the stress levels experienced by expectant mothers. women who perceive their spouse as supportive during pregnancy tend to experience a greater sense of adequacy and higher relationship satisfaction in the postpartum period. While, Powell and Karraker [16] emphasized that women feel more adequate postpartum period and higher relationship satisfaction when they sense a supportive spouse during pregnancy. Contrary, lacking support of the spouse during pregnancy has a negative association with the emotional well-being of wives, resulting in an increased risk of negative pregnancy outcomes and postpartum period [17]. Similarly, Bhuiya and Chowdhury [18] highlighted the lower chances of survival for children of divorced mothers compared to non-divorced mothers. Suresh and Balram [19] stressed the significance of educating and empowering males regarding pregnancy complications to improve maternal and neonatal health outcomes. spouses who have knowledge about complications during pregnancy and childbirth are more likely to actively support their wives in seeking antenatal care, opting for institutional deliveries, and receiving postnatal assistance. Their understanding of these complications plays a crucial role in promoting birth preparedness [20]. Furthermore, Spousal accompaniment during women’s health assistance is associated with increased utilization of skilled maternal health services, as reported by Rahman et al. [21] and Adeniran et al. [22]. It is recommended to implement special initiatives that encourage partners to accompany their wives and raise awareness about maternal health issues. Additionally, Yargawa and Leonardi-Bee [23] found that male involvement in pregnancy-related outcomes leads to a lower risk of postpartum depression and improved utilization of maternal health services. Women with supportive partners are more likely to receive skilled birth attendance and proper postnatal care. Mannion et al. [24] emphasized the significance of partner support in enhancing breastfeeding confidence and sustainability. Partner support, including vocal motivation and active participation in breastfeeding practices, plays a crucial role in empowering mothers in their breastfeeding journey. Furthermore, Mohammed et al. [25] emphasized the need for interventions that focus on enhancing partner support to improve pregnancy-related outcomes, such as reducing prenatal stress, depression, and smoking among pregnant women. Interventions targeting partner support can have a positive impact on pregnancy outcomes and contribute to better overall maternal and infant health. Overall, the literature suggests that partner support plays a crucial role in promoting maternal well-being and positive pregnancy outcomes, highlighting the need for interventions to enhance partner involvement and support during pregnancy.

### Research objectives

The National Institute of Population Studies (NIPS) executed a survey in 2019 intending to provide up-to-date estimates of essential demographic and health indicators. Particularly, the survey was designed and carried out to assess whether Pakistan stands on maternal health indicators and how well the country is moving toward these targets. In light of these goals, this study was conducted to achieve the following objectives:

- To assess the impact of partner support during pregnancy on maternal health.
- To asses the impact of partner support on postpartum problems
- To assess the impact of pregnancy related problems on safe child-birth.

## Research methodology

### Universe of the study

The sampling universe of 2019 survey includes the urban and rural regions of the four provinces of Pakistan (Punjab, Sindh, Khyber Pakhtunkhwa (KP), and Balochistan), Azad Jammu and Kashmir (AJK), and Gilgit Baltistan (GB). Restricted military and protected areas were excluded from the sample. Below is the details of important variables included in the study:

#### “Partner relationship” variable

Partner’s support may be particularly encouraged during antenatal care and potentially modifiable target to improve pregnancy outcomes. The survey collected data on a dichotomous variable Partner Relationship: pregnant women living with and away from their partner. Women living with their partners was the reference category. The class of pregnant women living away from their husbands consists of widowed, divorced, and legally separated women in Pakistan. Partner behavior refers to the actions, attitudes, and involvement of a woman’s spouse in relation to various aspects of her life, including health and well-being. In the context of this study, partner behavior may specifically refer to how a wife’s partner influences or impacts her health behaviors, decision-making, and maternal health outcomes.

#### Problems in pregnancy related outcomes

The women provided information on a range of prenatal health complications. These health complications were divided into three major categories i.e. problems during pregnancy, problems during labour and delivery, and problems during postpartum.

### Data source

The data for this study comes from the Pakistan Maternal Mortality Survey (PMMS) 2019 conducted by the NIPS under the umbrella of Ministry of National Health Services, Regulations and Coordination. Data collection was carried out between $20^{th}$ January and $30^{th}$ September 2019. ICF offered the technical assistance through DHS Program, and was funded by the United States Agency for International Development (USAID). Other agencies that facilitated the successful execution of the survey includes United Nations Population Fund (UNFPA), Department for International Development (DFID), and Bill and Melinda Gates Foundation. The PMMS used a multistage and multi-phase cluster sampling methodology based on the sampling frames derived from the $6^{th}$ Population and Housing Census, conducted by the Pakistan Bureau of Statistics (PBS) in 2017 [26]. The data is openly available on The Demographic and Health Surveys (DHS) Program and can be downloaded from the url http://dhsprogram.com.

### Missing cases

Generally, the missing rate was very low on all factors, including only 6.9% missing values in the Partner Relationship, 2.6% in age, and 2.9% in marital status variables. However, cultural barriers play a role in causing high non-response in pregnancy-related problems and pregnancy loss variables. The variables problems during pregnancy, problems during labour and delivery, problems in postpartum, and pregnancy loss have 52.5%, 57.6%, 52.5%, and 2.6% missing values, respectively. The pairwise deletion method was used in the analysis to overcome such a large number of missing cases. The pairwise deletion approach is suitable in situations where the statistical technique uses cases containing some missing observations. This procedure deletes the particular observation if the variable under consideration has a missing value. However, this observation can still be used in the analyses while analyzing other variables with non-missing values [27, 28].

### Statistical methods

#### Cluster analysis

In order to facilitate the process of data analysis, as a first step, descriptive techniques were implemented for data summarization. The tables and figures were used to describe the variables. Furthermore, the clustering approach was adopted to identify women facing similar problems during pregnancy and delivery. The clustering technique is an unsupervised learning problem that involves the designation of natural subgroups in a large dataset [29]. The goal of this stage was to identify women with similar health conditions. The features that were chosen for clustering of the dataset include Virginal Bleeding, Jaundice, Vomiting, Sever Headache, Weakness, Coma, Chest pain, Cough, High B.P, weight gain, Body aches, Swelling/feet, Swelling/face, Fits/Seizures, Fever, Lower Abdomen Pain, General Abdomen Pain, Blur vision, Shortness of Breath, Diabetes, Micturition, Puss in Urine, Anemia, and Weight Loss. Once the clusters were identified, profiling of each segment were carried out on the attributes, including women living with and away from husband, Region of residence, and age. Profiling each segment obtained as a result of cluster analysis will help identify problems that pregnant women suffer in the case of non-supportive male partners [30].

#### Clustering parameters

The clustering process was done using the *flexclust()* package in R-software [31]. The choice of parameters used in the cluster analysis was as follows:

- Algorithm: The *k-*Modes clustering technique was applied to the variables included in the study to identify the hidden patterns and natural sub-groups in the data. As most of the variables included in the study are categorical, so we decide the *k*-Modes algorithm instead of *k*-means. The portioning clustering was decided due to its speed and capacity to operate with large datasets.
- Number of clusters (*k*): The partitioning clustering algorithm requires the number of clusters as an input parameter. However, by nature, clustering is an unsupervised analysis process; therefore, the optimal number of clusters is case-dependent. In this article, we were not concerned about having equally sized clusters with clear dividing boundaries but rather clusters that represent different combinations of the clustering features in order to create user profiles with different interests and contexts. After some experimentation, we found that k = 3 clusters were suitable for our dataset.
- Similarity measure: One of the important parameter, while clustering a dataset, is to decide a distance function that can be used for computing the proximity between cases. We used the Gower Distance function, which is suitable for computing the distance between categorical variables.

#### Inductive statistical tests

In the second step, Inductive statistical tests including chi-square were perform to statistically test the association between various variables of interest. The chi-square test for independence likens two variables to determine if they are correlated [32]. The chi-square tests were conducted to explore the relationship between postpartum problems with Partner Relationship during pregnancy. Statistical significance was established at *p*-values of $< 0.05$.

#### Logistic regression

Finally, a bi-variate logistic regression model was used to associate the loss of pregnancy (Y) with the prominent problems during pregnancy and problems during labour (X). Logistic regression is a classification algorithm used to predict a binary outcome based on a set of independent variables [33–35]. In this section, we attempted to estimate the likelihood that a pregnancy would be lost (yes or no) due to various pregnancy and labor-related issues. Below is the mathematical description of the model used in this research article:

1 $$\begin{aligned} log\left( \frac{p}{1-p} \right) = \alpha + \beta \underline{X} \end{aligned}$$

where $p = \frac{1}{1+e^{-\left( \alpha +\beta \underline{X} \right) }}$ is the probability of the dependent variable Y being 1 given X i.e. the probability of pregnancy lost given problems during pregnancy features. These variables are chosen from the cluster profiling and significant factors obtained from the chi-square test. Table 1 below represents the variables along with their nature included in the logistic regression model.

**Table 1:** List of variables included in the logistic regression model

Variable	Meaning	Nature of Variable
Dependent (Y)	1-Pregnancy lost; 0-Pregnancy not lost	Dichotomous
Independent (X)		
Problems during pregnancy	Bleeding	categorical
	Jaundice	categorical
	Vomiting	categorical
	Headache	categorical
	Weakness	categorical
	Coma	categorical
	Chest pain	categorical
	Cough	categorical
	High B.P	categorical
	weight gain	categorical
	Body aches	categorical
	Swelling/feet	categorical
	Swelling/face	categorical
Problems during delivery	Prolong labor	categorical
	Bleeding	categorical
	Umbilical cord	categorical
	Baby premature	categorical
	Laceration	categorical
Suffered before pregnancy	High B.P	categorical
	Tuberculosis	categorical
	Varicose veins	categorical
	Severe anemia	categorical
	Kidney problem	categorical

## Results and discussion

### Reliability analysis

Cronbach’s Alpha is a convenient statistic used to assess a questionnaire’s reliability or internal consistency. In simple words, it provides an easy way to calculate if your score is reliable. As a general rule of thumb, Cronbach’s alpha coefficient of 0.70 or higher is appropriate, 0.80 or higher is more reasonable, and 0.90 or higher is optimal [36, 37]. The item’s Cronbach alpha factor for this study is 0.853, indicating that the item’s internal integrity is reasonably high.

### Descriptive statistics

Table 2 given below demonstrates the descriptive statistics of various variables. A total of 15,143 households (54.7% rural and 45.3% urban) were selected using a two-stage and two-phase cluster sampling approach. The survey was organised to provide estimates on maternal health indicators. Amongst them, 10.8% were currently pregnant, 0.5% were unsure, while 85.8% were not pregnant women. Only a small proportion of women (i.e. 41.6%) received antenatal care (ANC) during their last pregnancies. Similarly, just 20.5% of women received advice on early breastfeeding, 20.7% on exclusive breastfeeding, and 26.1% on a balanced diet during antenatal care. The importance of ANC in reducing maternal and newborn mortality cannot be overstated. However, in Pakistan, the utilization of ANC services is not well understood and remains relatively low. To address this issue and increase the uptake of ANC, initiatives should prioritize vulnerable and socially disadvantaged populations, such as rural, under-educated, and impoverished women. These groups often face barriers in accessing healthcare services and require targeted interventions to raise awareness about the importance of ANC [38]. Similarly, several studies have shown that a spouse’s educational level is positively linked with sufficient use of ANC services [39–42]. The significant chi-square result presented in Table 3 provides evidence to support and validate the observed relationship between spouse’s educational level and sufficient use of ANC services. Hence, it is crucial to implement awareness programs at the household level to improve the percentages of ANC service utilization. These numbers are depressing and contributes to Pakistan’s poor pregnancy outcomes, which are among the worst in the world, particularly when compared to other low-resource nations [43].

**Table 2:** Descriptive statistics

Variable	Category	Percentages
Area	Rural	57.7%
	Urban	45.3%
Pregnancy status	Yes	10.8%
	No	85.8%
	Unsure	0.5%
Antenatal care	Yes	41.6%
	No	58.4%
Early breastfeeding	Yes	20.5%
	No	79.5%
Exclusive breastfeeding	Yes	20.7%
	No	79.3%
Balance diet	Yes	26.1%
	No	73.9%

**Table 3:** Chi-square test for association between use of ANC and Spouse education level

Variable	Chi-square	*P*-value
Education level* Use of ANC	26.12	0.000*

**Fig. 1:**
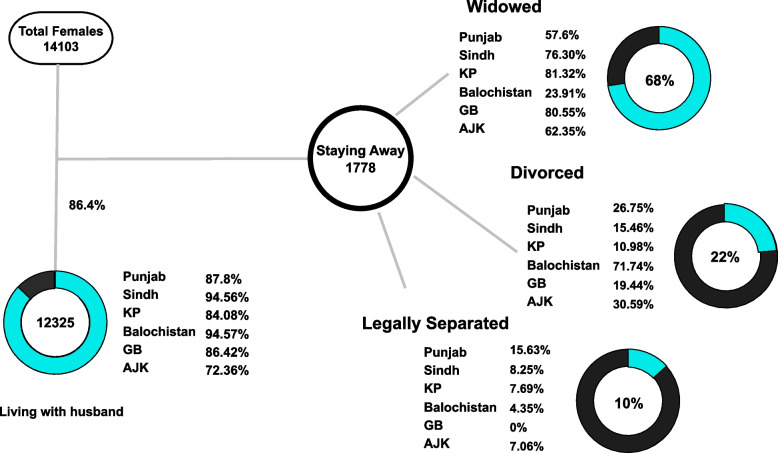
Province wise summary of women living with and away from their partners

As this study focused on partner relationships, Fig. 1 below demonstrates the province-wise summary of women living with and away from their partners. Balochistan has the least separation rate, where 94.57% of the women are living with their partners, followed by Sindh (94.56%), Punjab (87.80%), GB (86.40%), and KP (84.08%). The maximum separation rate is in AJK, where only 72.36% of women live with their partners. Among the group staying away from their partner during their last pregnancy, 68% were windowed, 22% were divorced, and 10% were legally separated. The separation rate was maximum in Balochistan (71.74% divorced and 4.35% legally separated) followed by AJK (30.59% divorced and 7.06% legally separated). This rate was minimum in KP (10.98% divorced and 7.69% legally separated) and Sindh (15.46% divorced and 8.25% legally separated). Punjab has a 26.75% divorce rate and a 15.63% legally separated rate. KP has the maximum percentage of widowed women (81.32%) amongst women living away from their partners, followed by GB (80.85%) and Sindh (76.30%). AJK and Punjab have 62.35% and 57.6% widowed women, respectively. In contrast, Balochistan has the minimum number of widowed population, comprising only 23.9% of the women living away from their partners.

### Cluster profiling

**Fig. 2:**
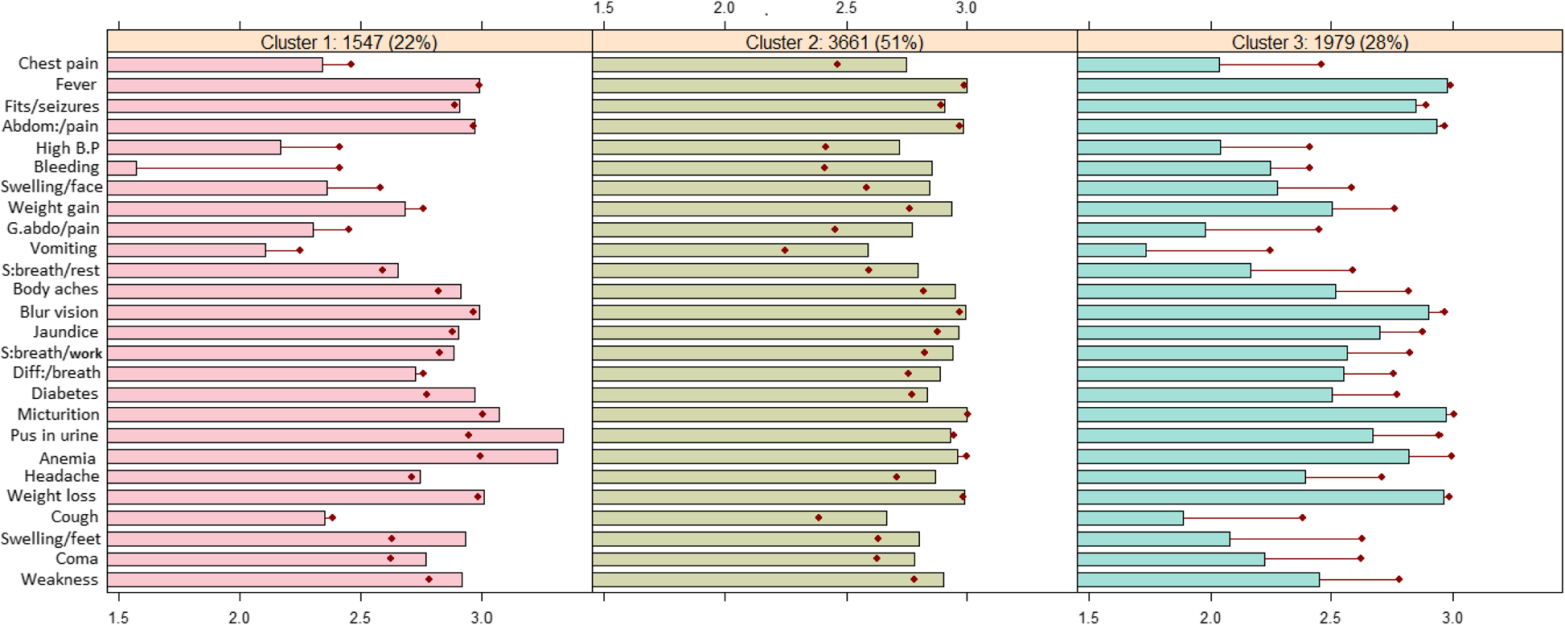
Profiling of clustering solution. The red dot in the Figure represents the overall mean for the corresponding variable, whereas the bar represents the within-cluster mean for the corresponding variable

The clusters from the dataset were produced using a traditional and standard method known as *k*-means algorithm. However, the hierarchical algorithm was used to demarcate the optimal number of clusters for the *k*-means algorithm. Based on the visual information of the dendrogram, different clustering solutions were generated using *k*-means cluster analysis. A comparison of these solutions indicated that the three-cluster solution was more compatible with achieving the study objectives. Figure 2 below demonstrates the detailed profiling of the cluster solution having three clusters.

- Cluster-2: The *k*-means Cluster-2 is the largest cluster comprising of 3661 (51% of entire data) cases. The substantial overall issues with pregnancy-related outcomes are seen in this cluster. During pregnancy, this group of women has a variety of health issues. The average for most of the variables for this segment of women is higher than the average for overall dataset. The average of variables Vaginal bleeding, Jaundice, Excessive vomiting, Headache, Sever weakness, Coma, Chest pain, Cough, High B.P, Weight gain, Body aches, Swelling of feet, and Swelling of face is higher for this segment of women. Theses factors are associated with a higher risk of many other complications, like preeclampsia, preterm delivery, and low birth weight [44]. Expectant mothers should always contact the doctor if they have any of these concerns during pregnancy.
- Cluster-3: The *k*-means cluster-3 is the second largest cluster comprises of 1979 (28% of all data) cases. This segment of women do not have much problems during pregnancies, as the cluster means for all variables are smaller than the overall means.
- Cluster-1: The *k*-means cluster-1 do not contribute much to the clustering solutions, as the within-cluster means of all variables are similar to the overall means of the dataset.

The variables Fever, Fits/Seizures, Lower abdomen pain, Weight loss, and Micturition do not contribute much to the data segmentation. Thus, the clustering approach separates the expecting mothers into two distinct groups, the first of which had medical conditions during their previous pregnancies and the second of which had minor complications.

The cluster membership by the variables “Women living together” and “Region of residence” is shown in Table 4 below. It is clear that the majority of women (70%) who live apart from their partner are members of the *k*-means cluster 2. As mentioned earlier, cluster 2 in the *k*-means clustering analysis represents a group of women who report ongoing health problems during their pregnancy. This finding serves as an important indicator that these women may face significant health challenges throughout their pregnancy, particularly if they do not have easy access to healthcare facilities or lack support from their spouse in seeking prenatal care. On the other hand, just 12% of the women in *k*-means cluster 3 indicated that they are living alone and are separated from their partners. Pregnant women in this group do not have any health complications. This finding suggests that women who live with their husbands or have strong and supportive relationships with them tend to have a higher likelihood of receiving adequate prenatal care and a lower probability of experiencing certain problems during pregnancy. These findings provide an important take-home message that high-quality family relationships have a positive impact on maternal health [45].

**Table 4:** Cluster wise summary of variables

Variable	Code	Cluster-1	Cluster-2	Cluster-3
Living together	Yes	1695	2522	1394
	No	292	1139	196
Region of residence	Punjab	499	1118	343
	Sindh	391	717	262
	KP	495	601	400
	Balochistan	246	528	204
	GB	141	314	189
	AJK	212	380	147

In the province-wise distribution, Punjab has the most significant proportion of women belonging to *k*-means cluster 2 followed by Balochistan and Sindh. In the Punjab province 1118 (57% of women in Punjab), in Balochistan 528 (53.9% of women in Balochistan), whereas in Sindh 717 (52.3%) belongs to this cluster. The proportion of women belonging to *k*-means cluster 2 is minimum in KP, where 601 (40.1% of women in KP) belongs to this particular cluster. This clearly indicates that mostly women in Punjab suffer problems during pregnancy. However, KP is the province where women receive antenatal care frequently, and the least number of women suffer health issues during pregnancy.

### Chi square results

Table 5 given below demonstrates the chi-square test results for testing the association between Partner Relationships and various health problems during the postpartum phase. The women constantly receiving advice from skilled attendants during the antenatal period is significantly associated with a healthy relationship. The three types of advice i.e Early breastfeeding ($p = 0.046$), Exclusive breastfeeding ($p = 0.041$), and Balance diet ($p = 0.033$) were significantly associated with a healthy relationship of the spouse. This is an indication that pregnant women who have a healthy relationship with their partners tend to receive better care during the antenatal period. This includes receiving proper nutrition, access to education about child and self-care, and sufficient information related to antenatal care. The findings were consistent with a study conducted by Nur et al. [46], who contends that the majority of postpartum mothers were ready and reminded to take their medications, eat healthy foods, and breastfeed their babies.

**Table 5:** Chi-square test for association of Partner Relationship with different variables

Variable	Code	Chi-square	*P*-value
During antenatal care advised on:	Early breastfeeding	6.14	0.046*
	Exclusive breastfeeding	6.56	0.041*
	Balanced diet	7.20	0.033*
Complications during postpartum	–	28.80	0.004**
Problems during postpartum:	Fever	7.289	0.608
	Seizures/fits	51.46	0.000**
	Excessive bleeding	25.34	0.007**
	Jaundice	4.10	0.900
	Vaginal discharge smelling material	16.56	0.031*
	Burning in micturition	2.85	0.970
	Increased frequency of urine	7.47	0.588
	Feeling extreme weakness	45.86	0.000**
	Pallor	20.88	0.011*
	Cough (difficulty in breathing)	6.04	0.735
	Breasts tenderness	16.93	0.048*
	Breast swelling	42.73	0.000**
	Breast infection	20.88	0.013*
	Tear/ulcer in breast	6.94	0.643

* Sig at 5% ** Sig at 1%

Similarly, complications during the postpartum period ($p = 0.004$) were significantly associated with a healthy relationship. This indicates that if a woman is living with her husband, she gets proper food and care during pregnancy and labour, and there is far less chance of complications during the postpartum period. The counsel includes encouraging women to clean wounds and refrain from sexual activity for a while and describing foods and beverages that might hasten wound healing, such as snake-head fish and herbal drinks [47]. On the other hand, if a woman doesn’t live with her husband during pregnancy or labour, she is exposed to various complications. Such as 8 problems out of 14 i.e Seizures/fits ($p = 0.000$), Excessive bleeding ($p = 0.007$), Vaginal discharge smelling material ($p = 0.031$), Feeling extreme weakness ($p = 0.000$), Pallor ($p = 0.011$), Breasts tenderness ($p = 0.048$), Breast swelling ($p = 0.000$), and Breast infection ($p = 0.013$) were significantly associated with Partner’s Relationship. When postpartum mothers lack support from their husbands, they may feel neglected and experience feelings of despair. Moreover, if postpartum anxiety is left unaddressed, it can lead to increased stress levels, which can manifest in negative attitudes and unfavorable behaviors. These behaviors may include a lack of appetite, a reluctance to monitor their health regularly, and potential harm to their overall well-being [48].

### logistic regression model

Table 6 below demonstrates the coefficient estimates of logistic regression model for safe-child birth as a function of problems during pregnancy and labor. Results of the models shows that eight of the problems during pregnancy i.e. Vaginal bleeding ($$p = 0.000$$), Excessive vomiting ($$p = 0.000$$), Chest pain ($$p = 0.013$$), Cough ($$p = 0.009$$), Excessive weight gain ($$p = 0.016$$), High B.P ($$p =.000**$$), Swelling/feet ($$p =.008$$), and Swelling/face ($$p =.046$$) were significantly associated with pregnancy lost. The results indicate that certain health symptoms during pregnancy are associated with an increased risk of miscarriage. Specifically, women who experience excessive vaginal bleeding are 2.262 times more likely to experience miscarriage, while those with excessive vomiting have a 1.885 times higher risk. Additionally, pregnant women with chest pain have a 1.178 times higher risk, cough has a 0.876 times risk, and high blood pressure has a 1.137 times risk of miscarriage. Excessive weight gain during pregnancy is also associated with a 0.910 times higher risk of miscarriage. These significant factors are related to women’s physical health and can be effectively managed and controlled during antenatal care. Regular visits to qualified healthcare professionals play a crucial role in addressing and monitoring these symptoms, thus reducing the risk of miscarriage. Therefore, in line with several research [49–51], mothers who have supportive husbands are more likely than their counterparts to have a safe delivery.

**Table 6:** Parameter estimation using logistic regression model of safe child-birth

Variable	Code	Coefficient	S.E	*P*-value	Exp(B)
Problems during pregnancy	Bleeding	.816	.063	.000**	2.262
	Jaundice	.149	.082	.069	1.161
	Vomiting	.122	.034	.000**	1.885
	Headache	.067	.039	.084	1.069
	Weakness	.105	.060	.080	1.111
	Coma	-.163	.098	.095	.849
	Chest pain	.164	.066	.013*	1.178
	Cough	-.133	.050	.009**	.876
	High B.P	.128	.034	.000**	1.137
	weight gain	-.095	.039	.016*	.910
	Body aches	-.042	.037	.263	.959
	Swelling/feet	-.136	.051	.008**	.873
	Swelling/face	.115	.058	.046*	1.122
Problems during delivery	Prolong labor	.161	.047	.001**	1.175
	Bleeding	.143	.063	.023*	1.153
	Umbilical cord	-.044	.031	.156	.957
	Baby premature	.312	.067	.000**	1.366
	Laceration	-.312	.062	.000**	.732
Suffered Before pregnancy	High B.P	.152	.070	.030*	1.164
	Tuberculosis	-.216	.125	.084	.805
	Varicose veins	.174	.098	.075	1.190
	Severe anemia	.180	.066	.007**	1.197
	Kidney problem	.205	.087	.018*	1.228
Constant	—	-.240	.432	.578	.786

* Sig at 5% ** Sig at 1%

Similarly, four of the problems during delivery and labor i.e. Prolonged labor ($p = 0.001$), Excessive bleeding ($p = 0.023$), Baby premature ($p =.000$), and Laceration ($p = 0.000$) were significantly associated with the miscarriage. Women who experience prolonged labor pain have a 1.175 times, while those with excessive bleeding have a 1.153 times higher risk of miscarriage. Additionally, women who give birth prematurely have a 1.366 times, and those who experience lacerations have a 0.732 times higher chance of miscarriage. these findings underscore the importance of comprehensive prenatal care that includes regular check-ups, proper monitoring, and timely interventions to identify and address any potential risks or complications. By effectively managing these factors, healthcare professionals can help reduce the likelihood of miscarriage and support better maternal and fetal health. Studies like [52] revealed that doctors must closely watch the baby’s heart rate and determine if the infant is tolerating labour. Being a caring spouse, the husband may offer rapid medical assistance in such situations, which can greatly enhance the partner’s safety.

Furthermore, three of the problems suffered before pregnancy i.e. High B.P ($p = 0.030$), Severe anemia ($p = 0.007$), and Kidney problem ($p = 0.018$) were significantly associated with miscarriage. Women having high B.P before pregnancy are 1.164 times, Severe anemia are 1.197, and kidney problems are 1.228 times more exposed to miscarriage. Additionally, the study refers to the research conducted by Irawan et al. [53], which demonstrated the positive relationship between husband’s support and the quality of life for postmenopausal women. Applying this understanding, it can be inferred that having a supportive husband can enhance the quality of life for women during the postmenopausal phase. This support may extend to the childbirth phase and contribute to a safer pregnancy experience, particularly for women with pre-existing health conditions.

## Conclusion

Pakistan’s position among the countries with poor pregnancy-related outcomes is a concerning issue, and it lags significantly behind many other nations. The exact reasons for these alarming maternal health situations in Pakistan have not been fully elucidated. However, this study aimed to investigate and shed light on the factors that may contribute to these disparities. The study utilized a representative sample of women collected from the PMMS conducted by the NIPS under the Ministry of National Health Services, Regulations, and Coordination. The PMMS survey was conducted between January $20^{th}$ and September $30^{th}$, 2019. The PMMS used a multistage and multi-phase cluster sampling methodology based on the sampling frames derived from the $6^{th}$ Population and Housing Census. The complete data is available for a total of 15,143 women participants over 18 years of age. Among them 2.9% have missing data on marital status, 52.5% on problems during pregnancy, 57.6% on problems during delivery and labor, 52.5% on problems during postpartum period, and 2.6% on miscarriage. To overcome such a large number of missing cases on certain attributes the case wise deletion method was used in the analysis.

In Pakistan, men take up the role of the women’s guardians by taking care of and meeting their financial needs. Males are expected to provide for the material needs of the family, while women are in charge of the children’s upbringing, education, grooming, and other requirements. In most nations, families base their health decisions on a variety of socioeconomic and cultural factors [54, 55]. Therefore, in Pakistan, the husbands decide everything pertaining to health. Most pregnant women did not obtain adequate treatment due to the male population’s lack of knowledge regarding the value of ANC in maternal health. Nevertheless, the use of ANC services in Pakistan needs to be better understood [56]. The study supports the theory that a husband’s level of engagement in a woman’s pregnancy, labor, and postpartum might influence her overall health, improve her conduct as a mother, and lessen her postpartum depression symptoms. It was discovered that pregnant women living alone experience several health issues, including bleeding, excessive vomiting, chest discomfort, coughing, high blood pressure, excessive weight gain, body pains, and swelling of the feet and face. Both inadequate prenatal care and lack of medical assistance during labour and delivery affect these women. They, therefore, also experienced medical issues during their postpartum period. Lack of prenatal care causes women who live away from their spouses to miscarry. These findings are crucial as no such study has been carried out in Pakitan that addresses the impact of the husband’s role during pregnancy on their spouse’s health. This can help policymakers to devise different education campaigns that encourage men to support their spouses throughout pregnancy and offer appropriate prenatal care.

## Limitations

This study is based on the dataset collected in the Maternal Mortality Survey 2019, which has certain limitations. These limitations are briefly discussed here.

- Missing cases: The dataset comprises a huge number of missing cases, especially on the variables related to women’s health problems. This is because the cultural restrictions played a role in the survey and resulted in a high rate of non-response on certain variables.

## Recommendations

 

- Awareness programs: Pregnant women and newborns require timely access to qualified health professionals during pregnancy, labour, and postpartum. However, their access to healthcare facilities is restrained by delays. The causes of these delays include logistical and financial concerns and inadequate community and family awareness and understanding regarding mother and infant health matters. So, there is a need to arrange awareness programs for male partners to fully support and provide proper antenatal care during and after pregnancies.
- Childcare guides: A supporting role for spouses can be included in the maternal and childcare guides provided during ANC visits and couple interventions at the community level.

## Data Availability

The data is openly available on The Demographic and Health Surveys (DHS) Program and can be downloaded from the url http://dhsprogram.com.
